# Profile of patients with post-tuberculosis bronchiectasis in a tertiary care hospital in Brazil

**DOI:** 10.1016/j.jctube.2022.100339

**Published:** 2022-11-13

**Authors:** Vitor Loureiro Dias, Mariane Gonçalves Martynychen Canan, Cleverson Alex Leitão, Elissa Ayumi Okuno, Giulia Rafaela Dainez de Sant'Ana, Juan Vitor Miranda

**Affiliations:** a Clinics Hospital of the Federal University of Paraná, Brazil; b Federal University of Paraná, Brazil

**Keywords:** Post-tuberculosis lung disease, Non-cystic fibrosis bronchiectasis, Tuberculosis

## Abstract

Bronchiectasis are abnormal permanently dilated bronchi which lead to chronic cough and other respiratory symptoms. Though tuberculosis (TB) is a common cause of bronchiectasis, data on this association are scarce. The objective of this study was to describe the profile of patients with post-TB bronchiectasis at a tertiary hospital in the southern region of Brazil. This was a retrospective study with data from patients in follow-up at our hospital from January 2005 to December 2020. We included patients 14 years of age or older who had bronchiectasis on chest computed tomography and a history of pulmonary TB. We excluded patients with bronchiectasis due to other causes or with confirmed non-tuberculous mycobacteria infection. We included 54 of the 204 non-cystic fibrosis bronchiectasis patients seen at our hospital during the study period. Most of the patients were female, older, and non-smokers. Less than a third had chronic bronchial infection by some agent. More than two thirds had some type of ventilatory defect, the most common being obstruction. More than half had upper-lobe impairment. Severity of the disease seemed to be equally distributed from mild to severe. Treatment was varied, including bronchodilators, inhaled corticosteroids, and azithromycin. We found that the profile of patients in our hospital is similar to that described in other studies, with slight differences in regard to microbiology and treatment.

## 1 Introduction

In 2020, about 10 million people fell ill with tuberculosis (TB) worldwide, making it a major cause of illness [1]. In Brazil, the incidence of TB was 31.6 cases/100,000 people in 2020, placing Brazil among the high-burden countries for TB and HIV-associated TB [2].

Post-TB lung disease is a heterogeneous condition characterized mainly by bronchiectasis, ranging from simple traction bronchiectasis to clinically significant bronchial disease. This form of bronchiectasis is poorly described in medical literature, and not much is known about how it relates to other forms of non-cystic fibrosis bronchiectasis (NCFB) [3].

In order to elucidate this gap in current knowledge about post-TB lung disease, we conducted an epidemiological study with patients in follow-up at the NCFB outpatient clinic of the Clinics Hospital of the Federal University of Paraná.

## 2 Methods

This was a retrospective study performed at the NCFB outpatient clinic of the Clinics Hospital of the Federal University of Paraná from January 2005 to December 2020, approved by the institution’s Committee for Ethics in Research on Human Beings under the opinion 28874720.0.0000.0096. We included patients 14 years of age or older who had bronchiectasis on chest computed tomography (CT) and a history of pulmonary TB. We excluded patients with bronchiectasis due to other causes (such as immunodeficiencies or autoimmune diseases) or with confirmed non-tuberculous mycobacteria infection. Bronchial infection was evaluated through periodic sputum samples, collected every 6 to 12 months. All chest CTs were analyzed by a single thoracic radiologist. Patients were classified as having mild, moderate or severe disease using the Bronchiectasis Severity Index (BSI), a score that combines clinical, radiological, and microbiological features. Treatment was categorized by medications in use at the last visit. Categorical variables were described as frequencies and percentages, and quantitative variables as means ± standard deviation or median (interquartile range [IQR]).

## 3 Results

During the study period, 204 NCFB patients were seen in our clinic, 54 of which met inclusion criteria (Table 1). Other causes of bronchiectasis were excluded. Our sample consisted mostly of female patients (n = 32, 59.3 %), with a mean age of 59.1 years. Most patients had no major risk factors for TB, except 5 (9.3 %) with diabetes, 4 (7.4 %) with HIV infection, 4 (7.4 %) with other types of immunosuppression and 1 (1.9 %) with chronic kidney disease. Moreover, 21 patients (38.9 %) had a history of smoking, of which only 2 (3.7 %) were current smokers.

**Table 1:** CHARACTERISTICS OF THE STUDIED POPULATION.

**Demographic and clinical characteristics [n = 54]**	
Female sex, n (%)	32 (59.3)
Age in years, mean ± SD	59.1 ± 13.8
BMI in kg/m^2^, mean ± SD [n = 44]	24.5 ± 4.7
Diabetes, n (%)	5 (9.3)
Chronic kidney disease, n (%)	1 (1.9)
HIV infection, n (%) [n = 37]	4 (10.8)
Non-HIV immunosuppression, n (%)	4 (7.4)
Current or former smokers, n (%)	21 (38.9)
Current smokers, n (%)	2 (3.7)
**Bacterial colonization [n = 41]**	
*Pseudomonas aeruginosa*, n (%)	5 (12.2)
Methicillin-susceptible *Staphylococcus aureus*, n (%)	1 (2.4)
Methicillin-resistant *Staphylococcus aureus*, n (%)	1 (2.4)
*Serratia marcescens*, n (%)	1 (2.4)
None, n (%)	33 (80.5)
**Functional characteristics [n = 45]**	
FVC in liters, mean ± SD	2.49 ± 0.97
FVC in % predicted, mean ± SD	75.7 ± 19.9
FEV1 in liters, median (minimum–maximum)	1.39 (0.58–3.78)
FEV1 in % predicted, median (minimum–maximum)	57.2 (26.8–135.4)
FEV1/FVC ratio, mean ± SD	59.7 ± 18.3
FEV1/FVC ratio in % predicted, mean ± SD	75.0 ± 23.3
Normal spirometry, n (%)	11 (24.4)
Obstructive ventilatory defect, n (%)	31 (68.9)
Response to bronchodilator, n (%) [n = 41]	7 (17.1)
**Predominance on chest tomography [n = 54]**	
Upper lobes, n (%)	31 (57.4)
Lower lobes, n (%)	7 (13.0)
Diffuse, n (%)	5 (9.3)
Unilateral, n (%)	11 (20.3)
**BSI, median (minimum**–**maximum) [n = 41]**	6 (0–15)
Mild bronchiectasis (0–4 points), n (%)	14 (34.1)
Moderate bronchiectasis (5–8 points), n (%)	13 (31.8)
Severe bronchiectasis (9 + points), n (%)	14 (34.1)
**Therapy [n = 54]**	
LABA/ICS combination, n (%)	40 (74.1)
LAMA, n (%)	6 (11.1)
LABA, n (%)	2 (3.7)
Azithromycin, n (%)	18 (33.3)
Supplemental oxygen, n (%)	4 (7.4)

ABBREVIATIONS: BSI = bronchiectasis severity index, ICS = inhaled corticosteroid, LABA = long-acting beta2-agonist, LAMA = long-acting muscarinic antagonist, SD = standard deviation.

Sputum samples were obtained from 41 patients (75.9 %). Chronic bronchial infection was found in only 8 patients (19.5 %), with identification of *Pseudomonas aeruginosa* in 5 of them (12.2 % overall, 62.5 % of those with positive cultures).

Of the 45 patients who underwent spirometry, 34 (75.6 %) had impaired lung function. The most common defect was obstruction, found in 31 patients (68.9 %), with an FEV_1_/FVC ratio of 59.7 ± 18.3, 75.0 %±23.3 % predicted, and a median FEV_1_ of 1.39 L (0.58–3.78 L), 57.2 % (26.8 %-135.4 %) predicted. Reduced FVC, either isolated or associated with obstruction, was found in 17 patients (37.8 %). In addition, 7 patients (17.1 % of those tested) had positive bronchodilator response. All patients had undergone chest CT, revealing a predominance of upper lobe impairment (n = 31, 57.4 %).

The Bronchiectasis Severity Score was assessed in 41 patients, and ranged from 0 to 15 points, with a median of 6 points. Of these 41 patients, 14 (34.1 %) were considered as having mild disease (0–4 points), 13 (31.8 %) as moderate (5–8 points) and 14 (34.1 %) as severe (9 or more points).

Regarding treatment, the most common choice was the association of long-acting beta_2_- agonists and inhaled corticosteroids prescribed to 40 patients (74.1 %), followed by six using long-acting muscarinic antagonists (11.1 %) and two using isolated long-acting beta_2_-agonists (3.7 %). Azithromycin was prescribed to 18 patients (33.3 %), of which 2 (11.1 %) had chronic infection by *P. aeruginosa*. Four patients had indication of long-term supplemental oxygen therapy (7.4 %).

## 4 Discussion

The demographic profile in our study was compatible with that of other studies. A predominance of women was also observed in patients with post-TB bronchiectasis in China [4] and in Saudi Arabia [5], as well as in patients with NCFB in Korea [6], in the United Kingdom [7] and in the United States [8]. The age range in these studies was also similar to ours, with a mean age of 56.8 years in China [4] and 68 in Saudi Arabia [5], and a median age of 55.1 years in Korea [6] and 61.8 years in the United Kingdom [7].

Our findings were slightly different from what is described in literature in regard to microbiology. Choi et al. [9] found no potentially pathogenic microorganisms in most patients with post-TB bronchiectasis and a predominance of *P. aeruginosa* in those with positive cultures, in agreement with our findings. However, they found different microorganisms in the remaining patients, with non-tuberculous mycobacteria in 13.6 %, *Enterobacteriaceae* in 4.2 % and *Haemophilus influenzae* in 1.7 %. Fong et al. [10], on the other hand, found positive cultures in about half of the studied patients, with non-tuberculous mycobacteria in 26.1 % of them, *P. aeruginosa* in 4.3 % and *Klebsiella pneumoniae* in 4.3 %.

The functional and tomographic characteristics of our sample were comparable to those described by other authors. The median FEV_1_ in our study (57.2 % predicted) was close to the ones found in studies from Saudi Arabia [5] (mean FEV_1_ of 62 % predicted), Korea [9] (median FEV_1_ of 57.6 % predicted) and Singapore [10] (median FEV1 of 63 % predicted). Regarding lesion distribution, Wang et al. [4] and Choi et al. [9] also found an upper-lobe predominance.

When analyzing lung disease severity, we found similar number of patients with mild, moderate, and severe disease, unlike other studies, which had a predominance of severe cases. Wang et al. [4] found that 40.5 % of patients had severe disease, while Al-Harbi et al. [5] found 60 % of severe patients.

Finally, we observed that most of the patients in our sample were using a combination of long acting beta_2_-agonists and inhaled corticosteroids. Though there is no indication to routinely use these medications in the treatment of these patients, Lee et al. [11] and Jeong et al. [12] have reported that bronchodilators lead to lung function improvement in patients with bronchiectasis. Choi et al. [9] found that patients with post-tuberculosis bronchiectasis have worse lung function and more tomographic findings in comparison to those with other NCFB, which could justify a more intensive approach to their treatment. Indeed, 68.9 % of our patients had obstructive ventilatory defect, a plausible indication for bronchodilator treatment. However, more studies are needed to assess the effect of these medications in these patients. The role of azithromycin is well-established for bronchiectasis patients with frequent exacerbations, especially in those infected by *P. aeruginosa* [13]. In our sample, azithromycin was prescribed to about a third of all patients, and to only 11.1 % of those with chronic *P. aeruginosa* infection. We did not find studies addressing the use of azithromycin in this specific group.

In conclusion, we found that the profile of patients in our hospital is similar to that described in other studies, with slight differences in regard to microbiology and treatment. Despite these similarities, there is a lack of consensus on the best treatment for these patients. Thus, more studies addressing this population are needed.

## CRediT authorship contribution statement

**Vitor Loureiro Dias:** Conceptualization, Investigation, Data curation, Formal analysis, Visualization, Methodology, Supervision, Writing – original draft, Writing – review & editing. **Mariane Gonçalves Martynychen Canan:** Conceptualization, Investigation, Visualization, Supervision, Writing – review & editing. **Cleverson Alex Leitão:** Investigation, Data curation, Formal analysis, Visualization, Methodology. **Elissa Ayumi Okuno:** Investigation, Data curation, Formal analysis, Visualization, Writing – original draft, Writing – review & editing. **Giulia Rafaela Dainez de Sant'Ana:** Investigation, Data curation, Formal analysis, Visualization, Writing – original draft, Writing – review & editing. **Juan Vitor Miranda:** Investigation, Data curation, Visualization.

## Declaration of Competing Interest

The authors declare that they have no known competing financial interests or personal relationships that could have appeared to influence the work reported in this paper.
